# Barley-ß-glucans reduce systemic inflammation, renal injury and aortic calcification through ADAM17 and neutral-sphingomyelinase2 inhibition

**DOI:** 10.1038/s41598-019-54306-8

**Published:** 2019-11-28

**Authors:** Maria Vittoria Arcidiacono, Natalia Carrillo-López, Sara Panizo, Anabel L. Castro-Grattoni, Petya Valcheva, Catalina Ulloa, Javier Rodríguez-Carrio, Anna Cardús, Covadonga Quirós-Caso, Laura Martínez-Arias, Carlos Martínez-Salgado, María José Motilva, Carmen Rodriguez-Suarez, Jorge B. Cannata-Andía, Adriana S. Dusso

**Affiliations:** 1 Division of Experimental Nephrology, IRBLleida, Lleida, Spain; 2 Bone and Mineral Research Unit, Hospital Universitario Central de Asturias, Instituto de Investigación Sanitaria del Principado de Asturias (ISPA), REDinREN-ISCIII, Oviedo, Spain; 3 Division of Nephrology, Hospital Universitario Central de Asturias, Oviedo, Spain; 4 Area of Immunology, Department of Functional Biology, Universidad de Oviedo, Oviedo, Spain; 5 Division of Infection & Immunity, School of Medicine, Cardiff University, Cardiff, Wales UK; 6 Department of Physiology and Pharmacology, University of Salamanca, IECSCYL, Instituto Biosanitario de Salamanca (IBSAL), REDinREN-ISCIII, Salamanca, Spain; 7 Department of Food Science and Technology, Agrotecnio Research Center, Lleida University, Lleida, Spain; 8 Departamento de Medicina, Universidad de Oviedo, Oviedo, Spain

**Keywords:** Nephrology, Phosphorus metabolism disorders

## Abstract

In chronic kidney disease (CKD), hyperphosphatemia-induced inflammation aggravates vascular calcification (VC) by increasing vascular smooth muscle cell (VSMC) osteogenic differentiation, ADAM17-induced renal and vascular injury, and TNFα-induction of neutral-sphingomyelinase2 (nSMase2) to release pro-calcifying exosomes. This study examined anti-inflammatory β-glucans efficacy at attenuating systemic inflammation in health, and renal and vascular injury favoring VC in hyperphosphatemic CKD. In healthy adults, dietary barley β-glucans (Bβglucans) reduced leukocyte superoxide production, inflammatory ADAM17, TNFα, nSMase2, and pro-aging/pro-inflammatory STING (Stimulator of interferon genes) gene expression without decreasing circulating inflammatory cytokines, except for γ-interferon. In hyperphosphatemic rat CKD, dietary Bβglucans reduced renal and aortic ADAM17-driven inflammation attenuating CKD-progression (higher GFR and lower serum creatinine, proteinuria, kidney inflammatory infiltration and nSMase2), and TNFα-driven increases in aortic nSMase2 and calcium deposition without improving mineral homeostasis. In VSMC, Bβglucans prevented LPS- or uremic serum-induced rapid increases in ADAM17, TNFα and nSMase2, and reduced the 13-fold higher calcium deposition induced by prolonged calcifying conditions by inhibiting osteogenic differentiation and increases in nSMase2 through Dectin1-independent actions involving Bβglucans internalization. Thus, dietary Bβglucans inhibit leukocyte superoxide production and leukocyte, renal and aortic ADAM17- and nSMase2 gene expression attenuating systemic inflammation in health, and renal injury and aortic calcification despite hyperphosphatemia in CKD.

## Introduction

In Chronic Kidney Disease (CKD), the development of hyperphosphatemia increases the risk of vascular calcification (VC) and cardiovascular mortality^1^. Elevations in serum phosphate (P), even to levels below the upper normal limit, increase the propensity for VC indirectly by worsening secondary hyperparathyroidism (SHPT) and bone de-mineralization^2^ and also directly, by inducing the osteogenic differentiation of vascular smooth muscle cells (VSMC)^3^.

Hyperphosphatemia also increases systemic inflammation^4,5^ and consequently, oxidative-stress-driven multi-organ injury^6,7^ worsening CKD-induced renal and vascular damage predisposing to VC and high mortality rates^1,3^. Part of the renal and vascular deleterious effects of oxidative stress involves the induction of two critical enzymes: ADAM17 (A Disintegrin And Metalloproteinase, also called TACE for Tumor necrosis factor Alpha Converting Enzyme)^8,9^ and neutral sphyngomyelinase 2 (nSMase2)^10^. Specifically, renal ADAM17 expression increases in CKD of all etiologies^11^ aggravating renal damage^12^ and systemic inflammation, the latter by releasing soluble TNFα to the circulation^9^. In turn, TNFα induces its own gene expression^13^, and also the ADAM17 gene^14^, generating a vicious ADAM17/TNFα feed-forward inflammatory loop that worsens multi-organ injury^9^. In fact, in the severe inflammation of LPS-induced endotoxic shock in mice, exclusive ablation of the ADAM17 gene in mouse myeloid cells is sufficient to markedly reduce mortality rates^15^.

In the vasculature, TNFα increases local inflammation aggravating VSMC osteogenic differentiation^7^, and also, nSMase2 gene expression and activity^16^, an essential determinant of aging-induced inflammation, atherosclerosis and VC^10,16,17^. In fact, nSMase2 gene ablation or pharmacological inhibition of nSMase2 activity markedly reduces age-enhanced inflammation in health^10^, the higher propensity for atherosclerotic lesions in the ApoE−/− mouse^17^ and, more significantly for CKD, the release of exosomes initiating medial calcification^16^.

Based upon current therapeutic limitations to lower hyperphosphatemia in CKD^18^, and considering that orally administered yeast ß-glucans efficaciously reduce multi-organ injury and mortality rates in LPS-challenged rats,^19^ this study was designed to examine whether an anti-inflammatory strategy with β-glucans could effectively attenuate systemic inflammation in health, including the mediator of early pro-senescent/pro-inflammatory interferon like responses, activated by the cGAS/STING pathway of cytosolic dsDNA recognition^20^ and also, renal and vascular injury predisposing to VC in a rat model of hyperphosphatemic rat CKD.

To test this hypothesis, among the multiple natural sources for β-glucans, we chose barley β-glucans (Bβglucans), based upon the safety of the FDA recommended daily intake of 3 to 5 g for their cholesterol^21,22^ lowering capacity. Specifically, we evaluated the efficacy of dietary Bβglucans to reduce: (a) Systemic inflammation in healthy adults with normal renal function; (b) Renal and aortic inflammation and the propensity for CKD progression and aortic calcium deposition in a rat model of hyperphosphatemic CKD, and (c) The contribution of Bβglucans regulation of ADAM17 and nSMase2 expression to their anti-inflammatory/anti-calcifying actions *in vivo* and *in vitro* in VSMC.

## Results

### Human study. Systemic anti-inflammatory actions by dietary BßGlucans

The daily intake of 3 g of BßGlucans, as barley bread by 10 individuals with normal renal function during 4 weeks, decreased circulating leukocyte mitochondrial superoxide production by 50% in one week (Supplementary Fig. 1).

**Figure 1:**
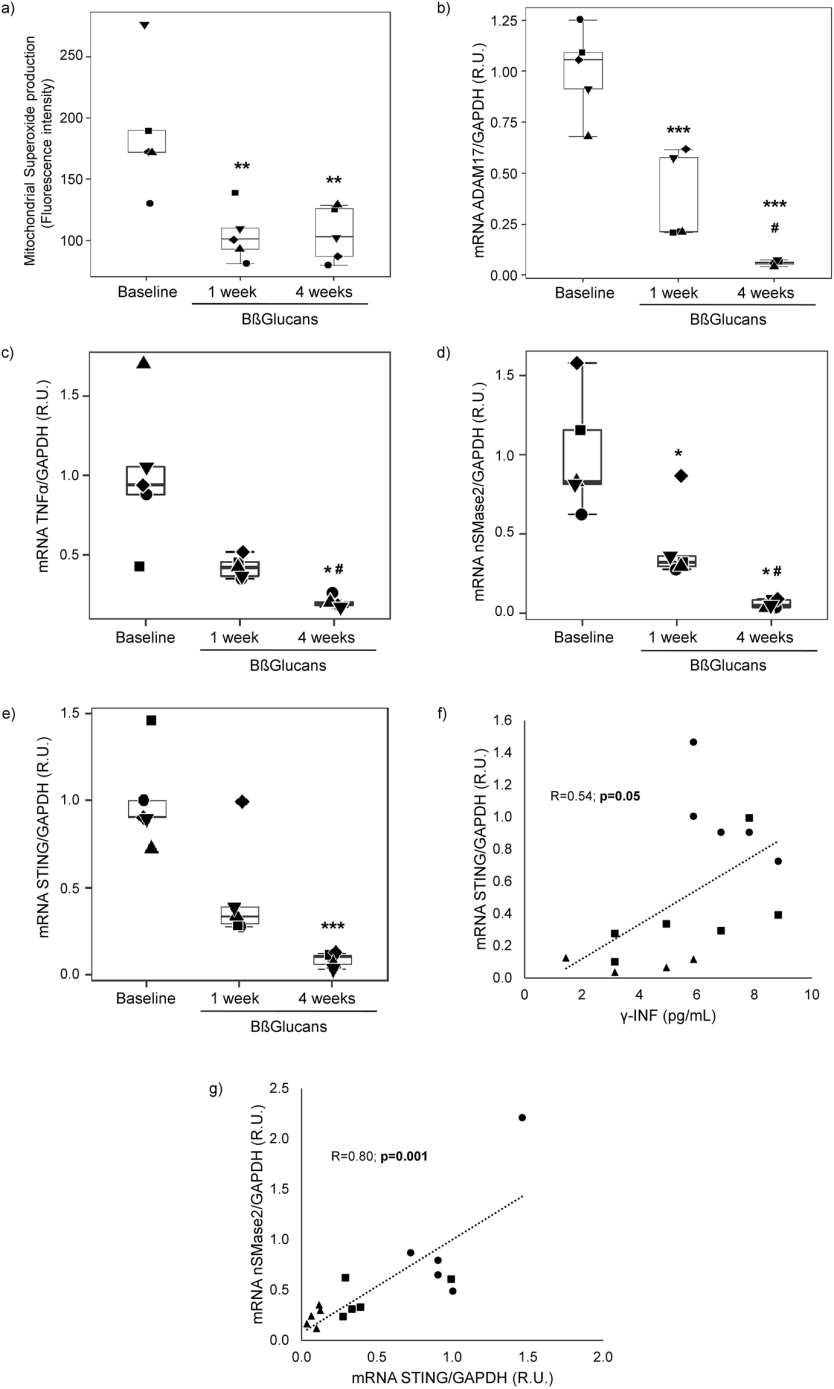
Dietary Bßglucans inhibit mitochondrial superoxide production, ADAM17, TNFα, nSMase2 and STING gene expression in circulating leukocytes from healthy adults. Box plot analyses of changes in baseline superoxide production (**a**), ADAM17 (**b**), TNFα (**c**), nSMase2 (**d**) and STING (**e**) mRNA levels (each shape represents one individual) in peripheral blood monocytes from 5 healthy adults ingesting 3 g of Bßglucans daily, as slices of barley bread, during 4 weeks. *p < 0.05 and **p < 0.01 vs. Baseline, ^#^p < 0.05 vs. 1week. Correlation between leukocyte STING mRNA and serum γ-interferon levels (f) and between leukocyte STING and nSMase2 mRNA levels (**g**). Each shape represent baseline (●), one week (■) and 4 weeks (▲) values.

In the 5 volunteers that completed the 3 g daily intake of BßGlucans for one month, the 50% inhibition of leukocyte superoxide production in the first week remained up to the end (week 4) (Fig. 1a). Furthermore, dietary BßGlucans also decreased leukocyte median mRNA levels of three recognized inflammatory markers: ADAM17 (80% at week 1 and 94% at week 4; p < 0.05) (Fig. 1b), TNFα (64% (not significant) at week 1 and 80% at week 4, p < 0.05) (Fig. 1c) and nSMase2 (58% at week 1 and 77% at week 4; p < 0.05) (Fig. 1d). These BßGlucans actions in circulating leukocytes after 1 or 4 weeks of a daily intake occurred despite no increases in serum levels either of β-glucans or in markers of bone and mineral homeostasis (calcium, phosphate, PTH, soluble klotho) or of systemic inflammation (C reactive protein and pro-inflammatory cytokines, except for a significant reduction of γ-interferon by week 4) (Table 1).

**Table 1:** Serum biochemistries in healthy volunteers.

	Baseline	1 week BßGlucans	4 weeks BßGlucans
	(n = 5)	(n = 5)	(n = 5)
**Serum ßglucans**			
(1,3)-β-D-glucan (pg/mL)	Undetectable	Undetectable	Undetectable
**Renal function**			
Creatinine (mg/dL)	0.86 ± 0.10	0.89 ± 0.12	0.90 ± 0.16
Urea (mg/dL)	23.9 [21.9–52.3]	26.3 [25.6–39.1]	26.8 [24.2–29.9]
Albumin (g/L)	46.02 ± 2.57	45.3 ± 1.48	44.46 ± 2.15
**Mineral homeostasis**			
Calcium (mmol/L)	2.37 ± 0.08	2.32 ± 0.07	2.32 ± 0.05
Phosphorus (mmol/L)	1.29 ± 0.18	1.18 ± 0.16	1.14 ± 0.23
PTH (pg/dL)	56.00 [36.40–58.40]	51.70 [33.00–55.90]	31.9 [30.9–43.2]
Klotho (pg/mL)	925.33 [809.83–1,003.17]	873.17[749.83–1,001.50]	868.00 [819.83–1,010.83]
**Inflammation**			
C Reactive Protein (mg/dL)	0.11 [0.04–0.22]	0.05 [0.04–0.10]	0.10 [0.04–0.13]
IL-2 (pg/mL)	1.37 ± 0.21	1.54 ± 0.26	1.47 ± 0.48
IL-6 (pg/mL)	4.31 ± 0.50	2.91 ± 1.52	3.55 ± 1.05
TNFα (pg/mL)	5.18 ± 3.15	5.20 ± 4.00	3.86 ± 1.47
γ-INFγ (pg/mL)	7.05 ± 1.28	6.31 ± 2.28	3.71 ± 1.73*
VCAM-1 (ng/mL)	2,180.68 ± 117.97	2,130.27 ± 34.16	2,246.75 ± 139.80

Values indicate Mean ± SD or Median (interquartile range). *p < 0.05 vs. Baseline.

Dietary BßGlucans-driven reductions in serum γ-interferon levels were paralleled by significant decreases in leukocyte STING mRNA (r = 0.54; p = 0.05; Fig. 1f). In fact, leukocyte STING mRNA levels decreased by 90% (p < 0.01) by week 4 (Fig. 1e) of dietary BßGlucans intake. Furthermore, this reduction strongly correlated with that of leukocyte nSMase2 gene expression (r = 0.80; p = 0.001; Fig. 1g).

### Animal study. Renal anti-inflammatory actions by dietary BßGlucans

**Figure 2:**
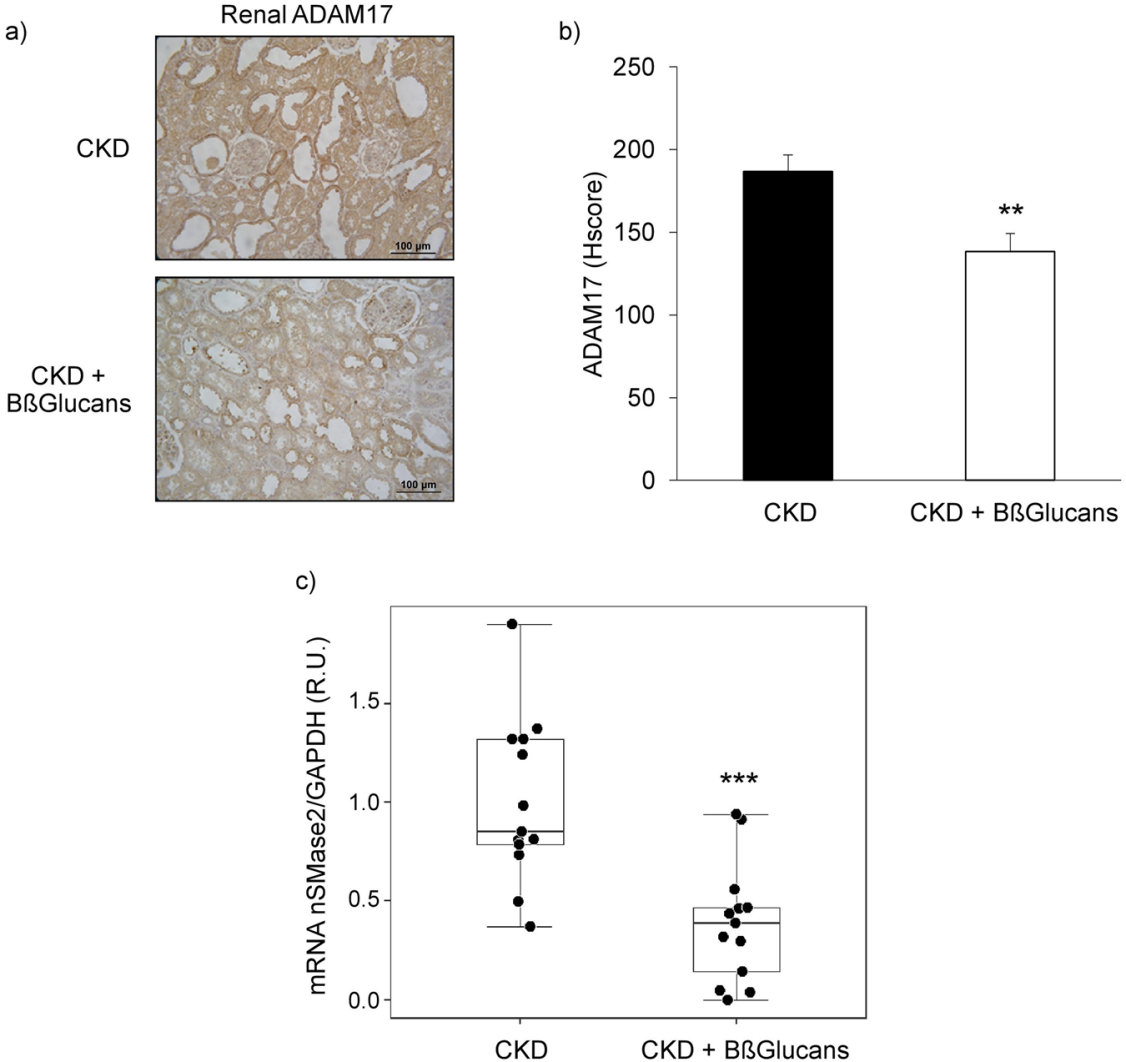
Renoprotection by dietary Bßglucans in hyperphosphatemic rat CKD. (**a**) Representative immunohistochemical images of renal ADAM17 from 5/6NX rats fed a high P diet with none (CKD; n = 13) or 2 mg of Bßglucans/g diet (CKD + Bßglucans; n = 13) during 4 weeks (Inset bars indicate relative scale); (**b**) Quantification of immunostaining. Bars and error bars represent mean ± SD of Histoscores values for all rats in both dietary groups; (**c**) Box plot analysis of nSMase2 gene expression in kidneys from rats described in (**a**). **p < 0.01 and ***p < 0.001 vs. CKD.

The administration of the high P diet containing BßGlucans to uremic rats had no effect on daily food consumption (20 g/day) or body weights (CKD: 234 ± 13 g to 248 ± 23 g; n = 13; CKD + Bßglucans: 237 ± 9 g to 255 ± 18 g; n = 13). Furthermore, despite undetectable serum (1,3)-ß-D-glucan levels, the CKD + Bßglucans group showed better renal function compared to the CKD group (lower serum creatinine, BUN, proteinuria and higher renal klotho) (Table 2), as well as reduced inflammatory markers (lower renal inflammatory cell infiltration (Table 2), ADAM17 protein and mRNA (Fig. 2a,b) and nSMase2 mRNA) (Fig. 2c). Serum and urinary TNFα levels were undetectable in both dietary groups.

**Table 2:** Biomarkers of renal dysfunction in rat CKD.

	CKD	CKD + BßGlucans	p value
	(n = 13)	(n = 13)	
Serum Creatinine (mg/dL)	1.0 ± 0.2	0.8 ± 0.2	**0.03**
BUN (mg/dL)	52.9 ± 9.6	44.3 ± 7.2	**0.02**
Phosphaturia (nmol/24 hours)	1.9 ± 0.5	2.3 ± 0.6	0.15
Proteinuria (mg/24 hours)	101.1 ± 80.0	21.4 ± 32.1	**0.04**
α-Klotho (IOD/Area)	1,477.2 ± 459.7	3,197.0 ± 1839.4	**0.02**
Lymphocyte infiltration (% Area)	2.2 ± 2.4	1.0 ± 0.7	**0.04**

Values indicate Mean ± SD.

Regarding bone and mineral parameters, there were no significant differences in serum calcium, phosphate, PTH, 25-hydroxyvitamin D, FGF23 and bone alkaline phosphatase between groups (Table 3). The higher renal klotho found in the CKD + Bßglucans group (Table 2) did not result in increases in 24 hours phosphaturia (Table 3).

**Table 3:** Serum biomarkers of bone and mineral metabolism in rat CKD.

	CKD	CKD + BßGlucans	p value
	(n = 13)	(n = 13)	
Calcium (mg/dL)	9.8 ± 0.5	9.2 ± 0.8	0.19
Phosphate (mg/dL)	8.1 ± 2.6	7.7 ± 2.2	0.66
PTH (pg/mL)	3107 ± 2152	2044 ± 1127	0.14
25-hydroxyvitamin D (ng/mL)	29.4 ± 8.7	27.9 ± 6.5	0.63
FGF23 (ng/mL)	10.9 ± 5.6	12.5 ± 6.5	0.58
Bone alkaline phosphatase (U/mL)	42.1 (33.3–70.2)	28.1 (16.9–74.0)	0.28
(1,3)-β-D-glucan (pg/mL)	Undetectable	Undetetectable	—

Values indicate Mean ± SD or Median (interquartile range).

### Animal study. Vascular anti-inflammatory actions by BßGlucans

**Figure 3:**
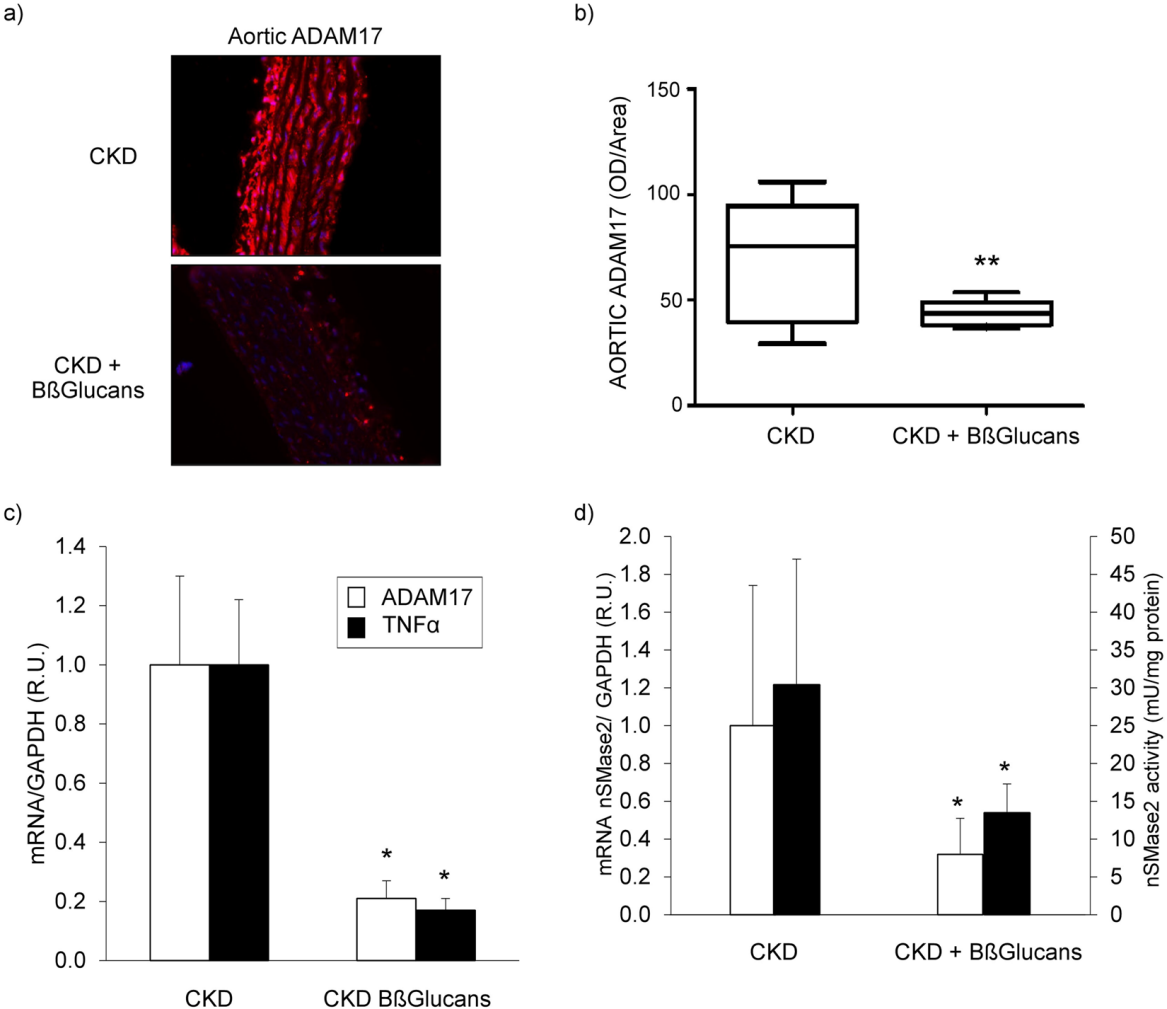
Anti-inflammatory vascular protection by dietary BßGlucans. (**a**) Representative immunostaining for ADAM17 (Inset bars indicate relative scale) in thoracic aortas from 5/6NX rats fed a high P diet with none (CKD; n = 13) or 2 mg of Bßglucans/g diet (CKD + Bßglucans; n = 13) during 4 weeks. (**b**) Quantification of aortic ADAM17 immunostaining. Box plots represent median (interquartile range) of OD/Area values for all rats in both dietary groups. (**c**) Aortic ADAM17 and TNFα gene expression; (**d**) Aortic nSMase2 mRNA (white bars; CKD n = 9; CKD + Bßglucans n = 7) and activity (black bars; CKD n = 13; CKD + Barley n = 11). R.U.: Relative units. Bars and error bars represent mean ± SD; *p < 0.05 vs. CKD.

The uremic rats fed the high P diet with Bßglucans had significantly lower median aortic ADAM17 protein (47%) (Fig. 3a,b), ADAM17 and TNFα mRNA levels (80%) (Fig. 3c) and nSMase2 mRNA levels (65%) and activity (55%) compared with the CKD group (Fig. 3d). Furthermore, in the aortas of these uremic rats, TNFα mRNA levels correlated directly with ADAM17 mRNA (r = 0.81; p < 0.001) and also with nSMase2 mRNA levels and activity (r = 0.89; p < 0.001 and r = 0.81; p < 0.01, respectively).

### Animal study. Vascular anti-calcifying actions by dietary BßGlucans

**Figure 4:**
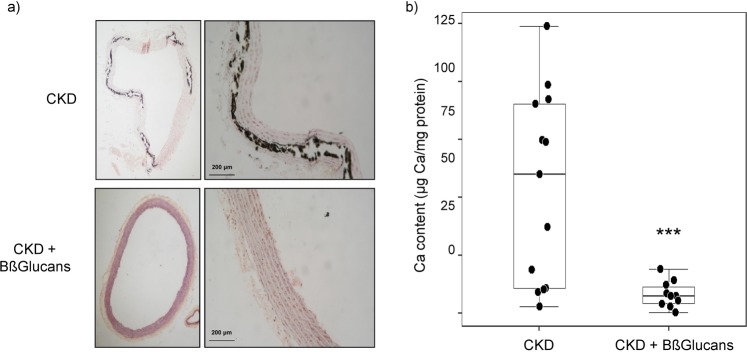
Anti-calcifying protection by dietary BßGlucans. (**a**) Representative calcium deposition measured by Von Kossa (black) staining (Inset bars indicate relative scale) in thoracic aortas from 5/6NX rats fed a high P diet with none (CKD; n = 13) or 2 mg of Bßglucans/g diet (CKD + Bßglucans; n = 13) during 4 weeks. (**b**) Quantification of calcium deposition in thoracic aortas described in (**a**); boxplot analysis of changes in calcium content, *p < 0.05 vs. CKD.

In the CKD group, 36% of the aortas were Von Kossa positive while all aortas from the CKD + Bßglucans group stained negatively (Fig. 4a). Furthermore, median aortic total calcium was 8 times lower in the CKD + Bßglucans group (7.5 µg Ca/mg protein) compared to uremic controls (CKD group: 60 µg Ca/mg protein) (Fig. 4b). Despite the higher Ca deposition found in the CKD group, there were minor changes in median aortic mRNA levels of osteogenic differentiation markers (reductions in α-actin and increases in Runx2 and Osterix) compared to the CKD + Bßglucans group, which did not reach statistical significance (Table 4). Instead, total aortic calcium content strongly correlated with increases in nSMase2 activity (r = 0.60; p < 0.01).

**Table 4:** Biomarkers of vascular osteogenic differentiation in rat CKD.

	CKD	CKD + BßGlucans	p value
α-actin mRNA (R.U.)	0.97 (0.69–1.45)	1.20 (0.37–1.41)	0.696
	N = 12	N = 9	
Runx2 mRNA (R.U.)	1.67 (1.11–2.58)	1.51 (0.9–1.91)	0.481
	N = 10	N = 11	
Osterix mRNA (R.U.)	0.72 (0.47–1.13)	0.53 (0.47–0.73)	0.374
	N = 12	N = 9	

Values indicate Median (interquartile range).

### *In vitro* studies. Direct anti-inflammatory/anti-calcifying actions by BßGlucans in VSMC

*Protocol 1* searched for an effective anti-inflammatory dose of BßGlucans extracts in the murine monocyte cell line Raw 264.7, exposed to 5 µg/mL of *E. Coli* LPS for 16 hours to reproduce severe systemic inflammatory stimuli.

Exposure to LPS increased mitochondrial superoxide production by 2-fold (Supplementary Fig. 1). A dose of 100 µg/mL of commercial BßGlucans extracts was necessary to fully prevent LPS-induced superoxide production. In unstimulated monocytes, the 100 µg/mL dose of BßGlucans extracts elicited anti-inflammatory actions as it reduced baseline monocyte superoxide production by 50% (Supplementary Fig. 1).

**Figure 5:**
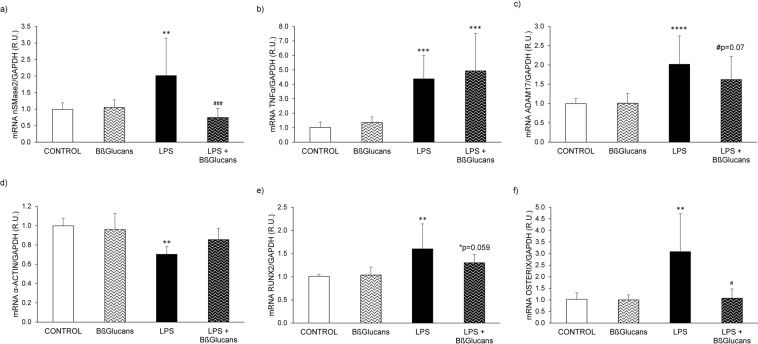
Bßglucans attenuate the vascular smooth muscle cell inflammation and osteogenic differentiation induced by LPS. Gene expression of nSMase2 (**a**), TNFα (**b**), ADAM17 (**c**), α-actin (**d**), Runx2 (**e**) and Osterix (**f**) in A7r5 cells exposed to control medium (CONTROL), 100 μg/mL of Bßglucans (β-GL), 5 µg/mL of LPS (LPS) or the combination of 100 μg/mL of Bßglucans and 5 µg/mL of LPS (LPS + ß-GL) for 16 hours. Bars and error bars represent mean ± SD from three independent experiments, each performed in triplicate per experimental condition. R.U.: relative units. **p < 0.01 and ***p < 0.001 vs. Control, ^#^p < 0.05 and ^###^p < 0.001 vs. LPS.

*Protocol 2* examined the impact of LPS-driven inflammation on VSMC phenotype. In A7r5 cells, the exposure to 100 µg of BßGlucans/mL for 16 hours had no effect on basal nSMase2, TNFα or ADAM17 gene expression (Fig. 5a–c). A7r5 exposure to LPS for 16 hours sufficed to significantly increase nSMase2, TNFα and ADAM17 gene expression (Fig. 5a–c) above the levels in unstimulated controls. Furthermore, the combination of the same dose of LPS with 100 µg/mL Bßglucans fully prevented LPS-driven increases in nSMase2 gene expression maintaining nSMase2 mRNA at the levels of the control group, failed to counteract LPS-induction of TNFα and decreased LPS-induced ADAM17 gene expression only marginally (Fig. 5a–c).

Regarding osteogenic differentiation, A7r5 exposure to 100 µg of BßGlucans/mL for 16 hours had no effect on basal mRNA levels of α-actin, Runx2 or osterix. Instead, while LPS exposure significantly decreased A7r5 levels of α-actin and increased Runx2 and Osterix gene expression compared to those in the control group (Fig. 5d–f), the combination of LPS with 100 µg/mL Bßglucans attenuated the stimulatory effect of LPS maintaining the osteogenic differentiation markers at levels similar to those in the control group (Fig. 5d–f).

**Figure 6:**
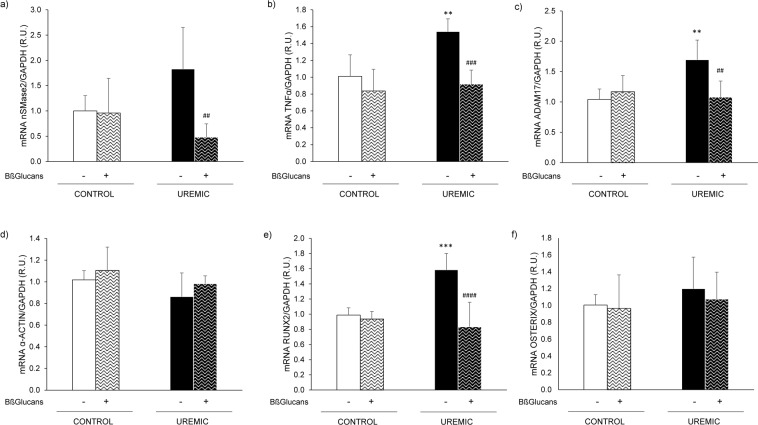
Bßglucans attenuate vascular smooth muscle cell inflammation and osteogenic differentiation induced by uremic conditions. Gene expression of nSMase2 (**a**), TNFα (**b**), ADAM17 (**c**), α-actin (**d**), Runx2 (**e**) and Osterix (**f**) in A7r5 cells exposed to serum from rats with normal renal function (Control), with or without 100 μg/mL of Bßglucans (β-GL), or exposed to serum from rats with 14 weeks of uremia fed a high phosphorus diet (Uremic) with or without 100 μg/mL of Bßglucans for 16 hours. Bars and error bars represent mean ± SD from three independent experiments, each performed in triplicate per experimental condition. R.U.: relative units. **p < 0.01 and ***p < 0.001 vs. Control, ^##^p < 0.01, ^###^p < 0.001 and ^####^p < 0.0001 vs. Uremic.

*Protocol 3* examined the impact of uremia-driven inflammation on VSMC phenotype. The exposure of A7r5 to uremic serum from hyperphosphatemic rats for 16 hours significantly increased TNFα and ADAM17 gene expression. However, the almost 2-fold increase in nSMase2 gene expression was not statistically significant (Fig. 6a–c). By contrast, exposure of A7r5 cells to uremic serum + Bßglucans maintained the mRNA of the three markers of inflammation at levels similar to those in the cells exposed to control serum, which was not affected by exposure to Bßglucans (Fig. 6a–c).

In A7r5 cells exposed to normal serum, Bßglucans had no effect on basal levels of the osteogenic markers. Instead, the uremic serum only increased Runx2 gene expression with no significant changes in either α-actin or Osterix (Fig. 6d–f). However, the addition of 100 µg/mL of Bßglucans to the uremic serum prevented the increases in Runx2 gene expression maintaining its levels at basal values.

### *In vitro* studies. Direct anti-calcifying and anti-nSMase2 actions by BßGlucans

**Figure 7:**
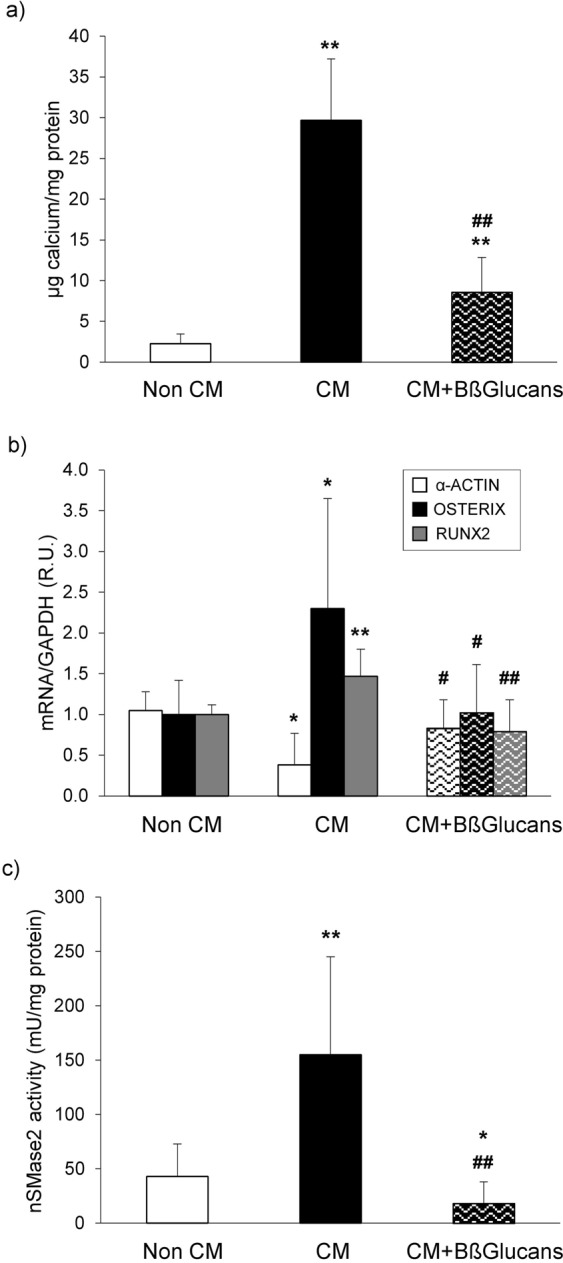
Bßglucans attenuate calcium deposition by inhibiting vascular smooth muscle cell osteogenic differentiation and nSMase2 activity. (**a**) Calcium deposition in A7r5 cells exposed to non-calcifying media (Non CM: 1 mM Ca; 1 mM P) or calcifying media (CM: 2 mM Ca; 3 mM P) with 0 or 100 μg/mL of Bßglucans (CM + β-GL) during 4 days. (**b**) Gene expression of α–actin (white bars), osterix (black bars) and Runx2 (gray bars) in A7r5 cells treated as described. (**c**) nSMase2 activity in A7r5 cells treated as described. Bars and error bars represent mean ± SD from three independent experiments performed in triplicate per experimental condition. R.U.: relative units. *p < 0.05 and **p < 0.01 vs. Non CM, ^#^p < 0.05 and ^##^p < 0.01 vs. CM.

In *Protocol 4*, A7r5 cells were exposed exclusively to calcifying media (CM: 2 mM Ca; 3 mM P) during 4 days, which increased calcium deposition by 13-fold. The addition of BßGlucans to the CM markedly attenuated (by 72%) the increases in calcium deposition induced by the CM (Fig. 7a), prevented the decrease in α-actin and the increases in osteogenic Osterix and Runx2 mRNA levels (Fig. 7b).

Similar results were found in *ex vivo* experiments culturing aortic rings from normal rats under the same calcifying conditions used in A7r5 (data not shown).

In addition, in A7r5 cells exposed to CM, the activity of nSMase2 increased by 3-fold, an induction totally prevented by adding 100 µg/mL of BßGlucans to the CM (Fig. 7c).

These direct anti-calcifying actions of BßGlucans extracts occurred in cells with undetectable mRNA levels of the β-glucan receptor Dectin 1 (not shown) and involved β-glucan internalization into A7r5 cells, as demonstrated by significant increases in intracellular BßGlucans levels from undetectable in cells exposed to the Non CM or CM alone to 2.3 ng/well (p < 0.01) in cells exposed to the CM + Bßglucans.

## Discussion

This work presents unprecedented properties of dietary Bβglucans of high translational relevance to attenuate systemic inflammation in health, as well as the progression of renal and vascular damage predisposing to VC in hyperphosphatemic rat CKD. These Bβglucans actions extend beyond the inhibition of leukocyte superoxide production and of STING/interferon-like pro-aging/proinflammatory signals that precede any elevation of systemic inflammatory markers above normal levels, and involve a marked suppression of leukocyte, renal and vascular ADAM17 and nSMase2.

### Bßglucans control of systemic inflammation in health

In healthy adults, the daily intake of 3 g of Bβglucans, recommended by the FDA to lower serum cholesterol^21,22^ could rapidly and efficaciously suppress systemic inflammation, a recognized inducer of VC. Indeed, dietary Bβglucans markedly reduced circulating leukocyte superoxide production (50%) as well as leukocyte ADAM17 and nSMase2 gene expression (60%) after a week intake, despite unchanged serum levels of CRP, TNFα and several other inflammatory cytokines, all within the normal range, and undetectable serum β-glucans concentrations. Despite the small size of this population of adults with normal renal function, Bβglucans rapid inhibition of these two pro-inflammatory genes in circulating leukocytes supports their potential to prevent/attenuate ADAM17-driven multi-organ injury and mortality rates^9^, and also nSMase2-driven age-enhanced inflammation regardless of serum TNFα^10^ and the propensity for atheromatous lesions^17^, as conclusively demonstrated in experimental models of ADAM17 or nSMase2 gene ablation.

Significantly, by week 4, dietary Bβglucans exerted a stronger (80 to 90%) inhibition of both ADAM17 and nSMase2 gene expression, and also markedly inhibited leukocyte TNFα mRNA levels, despite no further reductions of either of their inducers: superoxide production and serum TNFα. Thus, dietary Bβglucans inhibition of leukocyte ADAM17, nSMase2 and TNFα gene expression involved mechanisms other than suppressing oxidative stress induction of ADAM17^8,14^ and nSMase2 gene expression^10,23^, or TNFα-self up-regulation^13^. Significantly, among the inflammatory cytokines measured, dietary BßGlucans markedly reduced only circulating γ-interferon levels by week 4. This finding led us to evaluate whether dietary BßGlucans could also attenuate interferon-likepro-senescence/pro-inflammatory responses initiated by activation of the cGAS/STING pathway^20,24,25^. We found that serum γ-interferon not only strongly correlated with leukocyte STING mRNA but, more significantly, dietary BßGlucans caused a 90% reduction of leukocyte STING mRNA by week 4. Thus, leukocyte STING emerged as a novel sensitive biomarker of leukocyte pro-aging/pro-inflammatory features, as its reductions by dietary BßGlucans precede any elevations above normal in circulating levels of inflammatory cytokines, including γ-interferon. Furthermore, the strong correlation between leukocyte STING and nSMase2 mRNA levels suggests that dietary BßGlucans´progressive and simultaneous suppression of leukocyte STING and nSMase2 gene expression could contribute to attenuate the age-enhanced inflammation attributed to increases in nSMase2 in health. Indeed, enhanced nSMase2 release of exosomes from leukocytes carrying a cGAS/STING-driven senescence/inflammation-associated secretory phenotype would propagate these signals to healthy neighboring cells^26^. Even though the accuracy of these early biomarkers of the anti-aging, anti-inflammatory actions of BßGlucans was only identified in the 5 normal individuals who completed the 4 weeks of BßGlucans intake, the strength of the leukocyte STING/nSMase2 association (r^2^ = 0.65) provides a solid base for early antinflammatory actions that need to be further corroborated in larger cohorts and long-term studies.

### Bßglucans renal and vascular protection in hyperphosphatemic CKD

In hyperphosphatemic rat CKD, a daily intake of 40 mg of Bβglucans, as barley flour, effectively reduced inflammation-driven renal injury by targeting renal elevations in ADAM17, an enzyme induced by CKD of all etiologies^12^ and also by TNFα^14^ and oxidative stress^8^, despite undetectable serum β-glucan levels. The higher renal ADAM17 in uremic controls is sufficient to explain their increased inflammatory cell infiltration and 24 h proteinuria, as demonstrated by Lautrette and co-workers^12^. In turn, the higher proteinuria of uremic controls could mediate the faster decline in GFR, as recently reported^27^, and corroborated herein by the higher serum creatinine and BUN in the CKD group.

Dietary Bβglucans also reduced renal nSMase2 gene expression, which could extend the renoprotective benefits of this nutritional strategy to hypertensive individuals^28^, by counteracting angiotensin II-driven-increases in renal nSMase2 or to diabetic nephropathy, by reducing the release to the urine of megalin loaded exosomes^29^.

Bβglucans simultaneous attenuation of CKD progression and renal inflammatory cell infiltration, the two most potent down-regulators of renal α-klotho content^5,30^, resulted in a higher renal α-klotho in the CKD + Bβglucans group. However, phosphaturia was similar between dietary groups, which suggested that the higher renal klotho was insufficient to ameliorate kidney resistance to FGF23 and, consequently, the deleterious renal impact of phosphate retention.

In the inflamed aortas from these uremic rats, TNFα mRNA levels correlated directly with ADAM17 mRNA, partly reflecting the expected TNFα induction of ADAM17^9^ and of its own gene^13,14^, and also with nSMase2 mRNA levels and activity. Dietary Bβglucans suppressed by 80% the self-perpetuating aortic ADAM17/TNFα-inflammatory loop^9^ and prevented the 3-fold elevations in nSMase2 reported to prompt pro-calcifying exosome release^16^, thereby reducing aortic calcium deposition in the CKD + Bβglucans group by 8-fold compared to that in uremic controls. Interestingly, the higher calcium in uremic controls was unrelated to changes in the expression of markers of osteogenic differentiation and strongly correlated only with increased nSMase2. Thus, our aortic findings support the evidence that exosomes released by nSMase2 are the earliest calcified particles preceding overt calcification^31^, and also, that there are differential increases in vascular nSMase2 release of calcified and noncalcified exosomes causing heterogeneous calcium deposition at sites of early calcification^31,32^. Furthermore, Von Kossa staining was positive only in 36% of the aortas from uremic controls supporting that overt calcification is a late event in the course of CKD. Significantly, dietary Bßglucans fully prevented overt calcification, as none of the aortas in the barley group was Von Kossa positive despite no amelioration of systemic calcium and phosphate homeostasis.

Importantly, a Bβglucans intake effective at reducing aortic nSMase2 gene expression and activity had no adverse impact on bone mineralization, as estimated by serum bone specific alkaline phosphatase levels. This is an important translational consideration to design therapeutic anti-nSMase2 strategies in CKD because the nSMase2 null mouse presents severe bone and dental mineralization defects^33,34^.

### Mechanisms for Bßglucans´ actions

An intriguing aspect of Bβglucans potent anti-inflammatory, anti-ADAM17, anti-nSMase2, renal and vascular protective actions *in vivo*, is that they occurred with undetectable serum βglucans levels. Indeed, part of these actions may not require intestinal Bβglucans absorption because oral Bβglucans induce a saccharolytic shift in the microbiota that augments serum levels of anti-inflammatory short chain fatty acids, and also reduces circulating levels of p-cresyl-sulfate^35^, a uremic toxin promoting renal and cardiovascular injury^36,37^.

However, our studies *in vitro* in murine monocytes and VSMC exposed to commercially available Bβglucans extracts (95% purity) demonstrated direct anti-inflammatory, anti-calcifying actions that support the pharmacokinetics of dietary Bβglucans absorbed in the gut^38–40^. Specifically, in murine monocytes, we first identified that a dose of 100 µg/mL of Bβglucans extracts was necessary to prevent the 2-fold increases in mitochondrial superoxide production induced by a 16 h exposure to an LPS challenge (5 µg/mL). Significantly, this anti-inflammatory dose of Bβglucans also reduced by 50% the superoxide production in unstimulated monocytes, thus reproducing the 50% inhibition exerted in normal subjects after a week, or a month, of a daily intake of 3 g of Bβglucans.

Furthermore, the exposure of A7r5 cells to LPS (5 µg/mL for 16 h) corroborated that severe inflammation rapidly induced a switch to an osteogenic phenotype, as the increases in the mRNA levels for ADAM17, nSMase2 and TNFα were associated to decreases in α-actin and increases in the osteogenic markers Runx2 and osterix. Importantly, the simultaneous exposure of A7r5 cells to LPS and the effective anti-inflammatory dose of Bβglucans in immune cells, fully prevented LPS-induced inflammatory and osteogenic phenotypic changes without affecting VSMC baseline phenotype.

A7r5 cells also responded rapidly to uremic stimuli (serum from hyperphosphatemic rat CKD for 16 h) with significant elevations in ADAM17, TNFα and Runx2, but without significant increases in nSMase2 and osterix or decreases in α-actin. Nonetheless, Bβglucans fully prevented all uremia-induced changes, thus maintaining baseline levels of all of these inflammatory/osteogenic markers. These findings support that a threshold for inflammatory or uremic stimuli is necessary to increase nSMase2 and osteogenic markers to levels resulting in overt calcification.

In VSMC exposed exclusively to prolonged (4 days) high Ca/high P conditions, Bβglucans extracts decreased by 60% nSMase2 gene expression in the absence of any inflammatory or uremic stimuli, through a process involving β1,3-glucan internalization into VSMC that is Dectin1-independent, as A7r5 cells showed undetectable Dectin1 mRNA levels. Several β-glucans receptors expressed in VSMC (Complement Receptor 3 or TLR2/6)^41,42^ could mediate Bβglucans actions in A7r5 cells.

The internalization of (1,3)β-D-glucans into A7r5 cells exposed to Bβglucans extracts may also occur in other Bβglucans target cells, which could explain in part the systemic, renal and vascular anti-inflammatory/anti-nSMase2 actions of dietary Bβglucans in healthy adults and in hyperphosphatemic rat CKD despite undetectable serum levels. Indeed, there is evidence that soluble β1,3-glucose polymers, generated from oat and barley grains by the highly predominant β1,3-β1,4-glucanase in human gut bacteria^43^, are internalized and further processed by intestinal epithelial and immune cells to shorter β1,3-glucans. In turn, the shorter β1,3-glucans released to the circulation^39^ can be re-internalized into immune cells reducing serum levels while enhancing leukocyte systemic anti-oxidant/anti-inflammatory capacity^41,44^, as demonstrated herein in healthy adults.

Barley is the least expensive cereal grain. Therefore, dietary Bβglucans health benefits could reach the poorest populations of CKD patients and of healthy adults with systemic pro-aging or inflammatory problems and, therefore, at a higher risk for renal and vascular injury.

Prospective clinical trials are mandatory to evaluate the sensitivity and accuracy of leukocyte ADAM17, nSMase2 or TNFα mRNA and STING levels to personalize dietary Bβglucans interventions for efficacious systemic, renal and vascular protection in the general population and to attenuate CKD progression and the initiation of medial calcium deposition in human CKD.

## Material and Methods

### Ethics statements

Approval for the human study was obtained from the Institutional Review Board (Comité de Ética en Investigación Clínica del Hospital Universitario Central de Asturias), in compliance with the Declaration of Helsinki. All participants gave a written informed consent prior to their inclusion in the study.

Approval for the animal study was obtained from the Ethics Committee for Animal Experimentation at Lleida University in compliance with current international legislation for animal research.

All methods in the experimental protocols described below were carried out following the regulations for biomedical research of the University of Lleida and the University of Oviedo.

### Human study

Ten volunteers (2 men, 8 women, age 20 to 60 y.o.), with normal renal function ingested daily during 4 weeks barley bread, manufactured with flour from barley grains selected for their high (1-3)(1-4)-β-D-glucans content (8%), to provide 3 g of BßGlucans (produced at Food Science and Technology Department, Lleida University). Blood (5 mL) was drawn at baseline and at week 1 and 4 to obtain plasma and circulating leukocytes (after red cell lysis). As systemic inflammation biomarkers, we measured leukocyte mitochondrial superoxide production by flow cytometry in freshly isolated leukocytes. In the five volunteers (1 man, 4 women) that complied with the daily intake for one-month, we also quantified the mRNA levels of TNFα, ADAM17, nSMase2 and STING, as described in the *in vitro* studies. Serum levels of (1, 3)β-Dglucans, cholesterol, glycemia, markers of renal function, calcium and phosphate homeostasis, and systemic inflammation were measured as specified in blood chemistries. The reason to abandon the study was the omission of the daily intake more than twice per week.

### Animal study

Five-sixth nephrectomized (NX) female Sprague-Dawley rats (200–225 g) were fed during 4 weeks a high phosphate diet (HPD: 0.9% P; 0.6% calcium, Ca; Altromin) containing either 0 (CKD: n = 13) or 2 mg of BßGlucans/g diet (CKD + BßGlucans) from the barley flour described above, with no changes in dietary protein, P, carbohydrate or lipid content. The modified Megazyme method quantified final β-D-glucan content in the diet^45^.

### *In vitro* studies

I. BßGlucans anti-inflammatory actions were examined in the murine monocyte cell line Raw 264.7 and in the rat aortic VSMC line A7r5.
  *Protocol 1*: Rested Raw264.7, synchronized at G0 by exposure to FBS-free DMEM medium for 6 h, were treated for 16 hours with 5 μg/mL LPS from *Escherichia coli* 0111:B4 (L4391, Sigma-Aldrich), 100 μg/mL of barley (1–3)(1–4)-β-D-glucans (G6513, Sigma-Aldrich; 95% purity) or both.
  *Protocol 2*: A7r5 cells were exposed to 5 μg/mL LPS, 100 μg/mL of barley (1–3)(1–4)-β-D-glucans or both in DMEM + 1% foetal bovine serum (FBS) for 16 hours.
  *Protocol 3*: To test uremia-driven inflammatory stimuli, A7r5 cells were exposed to either control serum (a pool from rats with normal renal function fed a normal P diet; DMEM + 10% control serum), or to uremic serum (a pool from rats with uremia of 14 weeks fed a high phosphorus diet; DMEM + 10% uremic serum), with or without 100 μg/mL of Bßglucans for 16 hours.
II. BβGlucans anti-calcifying actions were examined in A7r5 cells and in aortic rings (1–2 mm) from normal rats.

*Protocol 4*: Aortic rings were washed in cold PBS containing P/S and then placed in fibronectin pre-coated (100 µg/mL) 6-well plates (8 rings/well) with growing media. A7r5 and aortic rings were exposed during 4 days to either non-calcifying medium (Non CM: DMEM-F12 + 0.1%BSA, 1 mM Ca, 1 mM P) or to calcifying medium (CM: DMEM-F12 + 0.1% BSA, 2 mM Ca, 3 mM P) with 0 or 100 μg/mL of BßGlucans. A7r5 levels of the β-glucan receptor Dectin1 were examined by qPCR and intracellular levels of (1–3) β-D-glucans as indicated in blood chemistries.

### Blood chemistries and proteinuria

Human biochemical parameters were measured at the Medicine Laboratory of the Hospital Universitario Central de Asturias using Cobas 8000 (Roche Diagnostics) Module c702, for most parameters, and Module e801 for PTH immunoassay.

For animal studies spectrophotometry and immunoassay (Cobas 8000, Roche Diagnostics) were used to measure serum levels of Ca, P, creatinine and 25-hydroxyvitaminD (25(OH)D). QuantiChromTM Urea Assay Kit measured Blood Urea Nitrogen (BUN, BioAssay System). ELISA Kits were used to measure rat intact PTH (Immutopics), rat fibroblast growth factor 23 (FGF23; EMD Millipore), rat bone alkaline phosphatase (Biosource), blood and urinary rat TNFα (Abcam plc, Cambridge, UK). Serum human TNFα was measured by bead-based multiplex assay (BiolegendPlex, Biolegend, Germany) analyzed in a FACS Canto II flow cytometer (BD Biosciences) equipped with a FACS Diva 6.5 software, following manufacturers´ protocols. Test strips measured urinary protein (SIEMENS MULTISTICK 10SG, Analyticon Biochemistry). The FungitelTM kit (Associates of Cape Code, Inc) measured serum, plasma and intracellular (1,3)β-D-glucans.

### Histological analyses

Rat renal and aortic 5 µm-paraffin sections were deparaffinized and hydrated. Renal ADAM17 and CD45 (leukocyte infiltration) immunostainings were performed as in^46^ and renal α-klotho with kit-CTS008 (R&D System). For aortic ADAM17 immunofluorescence nuclei were counterstained with Hoechst. For renal ADAM17, sections were counterstained with hematoxylin-eosin. Each slide had its negative control (no primary antibody). Supplementary Table 1 lists primary antibodies and dilutions. Quantifications used ImageJ or Histoscores^47^.

### Mitochondrial superoxide production

Circulating human leukocytes and cultured Raw 264.7 cells were washed with PBS, resuspended in 5 µM superoxide sensitive probe MitoSOX^TM^ (Thermo-Fisher Scientific) in PBS with either vehicle or the corresponding treatment for 10 min at 37 °C, protected from light and washed twice with PBS. A FACS Canto^TM^II flow cytometer (Becton Dickinson Bioscience) measured superoxide anion levels.

### Von kossa staining

Deparaffinized, hydrated rat aorta sections were incubated in 5% silver nitrate before the revealing solution. Slides placed in 2% sodium thiosulfate were counterstained with nuclear fast red.

### Total calcium measurements

A 20 mm segment of the abdominal rat aorta proximal to the iliac bifurcation was first grinded in liquid nitrogen and then decalcified with 0.6 N HCl. A7r5 cells, washed thrice with PBS, were decalcified with 0.6 N HCl.

Samples were shaken gently at 4 °C for 24 h. Upon centrifugation, O-cresolphthalein-complexone measured total calcium in supernatants. Pellets were re-suspended in lysis buffer (0.1 N NaOH, 0.1% SDS) for protein extraction and quantification (Lowry method, Bio-Rad).

For rat aortic rings, total calcium was measured as above in cell pellets from RNA extraction (below) and expressed as µg calcium/µg RNA.

### Sphingomyelinase2 activity

The Amplex Red Sphingomyelinase assay kit (Molecular Probes, Invitrogen) was used in aorta sections and in A7r5 whole cell extracts.

### Quantitative PCR

Total tissue or cell RNA, extracted with TRI reagent (Sigma-Aldrich), was reversed transcribed with a High-Capacity cDNA Reverse Transcription Kit (Applied Biosystems). Quantitative-real time PCR (qPCR) reactions used the Stratagene Mx3005P QPCR System (Agilent Technologies), Fast Start Universal Probe Master (Roche), pre-developed assays (Thermo-Fisher Scientific) and ΔΔCt quantification^48^.

### Statistical analysis

T tests, ANOVA or Kruskal-Wallis with Bonferroni post-hoc test examined statistical differences between groups. Results are expressed as mean ± SD unless otherwise stated. Statistical analyses used GraphPad Prism, SPSS 17.0 for Windows or R.

## Supplementary information

Supplementary information

## Data Availability

No datasets were generated or analyzed during the current study.
