# Supplementary material for: Barley-ß-glucans reduce systemic inflammation, renal injury and aortic calcification through ADAM17 and neutral-sphingomyelinase2 inhibition
Source: Sci Rep. 2019 Nov 28;9:17810. doi: 10.1038/s41598-019-54306-8 (PMC6882851; doi:10.1038/s41598-019-54306-8)

Barley-ß-glucans reduce systemic inflammation, renal injury and aortic calcification through ADAM17 and neutral-sphingomyelinase2 inhibition.

Authors and affiliations: Maria Vittoria Arcidiacono, PhD[1,*]; Natalia Carrillo-López, PhD[2*]; Sara Panizo, PhD[2*]; Anabel L. Castro-Grattoni, MS[1^]; Petya Valcheva, PhD[1^]; Catalina Ulloa, MD[3^]; Javier Rodríguez-Carrio, PhD[2,4]; Anna Cardús, PhD[1,5]; Covadonga Quirós-Caso, PhD[4]; Laura Martínez-Arias, MS[4]; Carlos Martínez-Salgado, PhD[6]; María José Motilva, PhD[7]; Carmen Rodriguez-Suarez, MD[5], Jorge B. Cannata-Andía, MD, PhD[2,8#] and Adriana S. Dusso, PhD[1,2#].

[1]*Division of Experimental Nephrology, IRBLleida, Lleida, Spain.*
[2]*Bone and Mineral Research Unit, Hospital Universitario Central de Asturias, Instituto de Investigación Sanitaria del Principado de Asturias (ISPA), REDinREN-ISCIII, Oviedo, Spain.*
[3]*Division of Nephrology, Hospital Universitario Central de Asturias. Oviedo, Spain.*
[4]*Area of Immunology, Department of Functional Biology, Universidad de Oviedo, Oviedo, Spain.*
5Division of Infection & Immunity, School of Medicine, Cardiff University, Cardiff, Wales, UK.
[6]*Department of Physiology and Pharmacology, University of Salamanca, IECSCYL, Instituto Biosanitario de Salamanca (IBSAL), REDinREN-ISCIII, Salamanca, Spain.*
[7]*Department of Food Science and Technology, Agrotecnio Research Center, Lleida University, Lleida, Spain.*
[8]*Departamento de Medicina, Universidad de Oviedo, Oviedo, Spain.*

*Equal contribution as first authors
^Equal contribution as second authors
[#]Equal contribution as senior authors

Supplementary Table 1. Primary antibodies and dilutions used for immunohistochemistry (IHC) or immunofluorescence (IF).

Primary antibody	Dilution	Technique	Comercial
Rabbit anti-ADAM17	1:400	IHC	EMD Millipore (#Ab19027)
Rabbit anti-ADAM17	1:100	IF	EMD Millipore (#Ab19027)
Rabbit anti-CD45	1:50	IHC	Abcam plc (#ab10558)
Mouse anti-Klotho	1:100	IHC	R&D Systems (#AF1819)

Supplementary Figure 1. Dietary Bßglucans inhibit mitochondrial superoxide production in circulating leukocytes from healthy adults. Box plot analyses of changes in baseline superoxide production in peripheral blood leukocytes from 10 healthy adults ingesting 3 g of Bßglucans daily, as slices of barley bread, during 4 weeks. *p<0.05 and **p<0.01 vs. Baseline, #p<0.05 vs. 1week. White circles mark the 5 patients that complied with the 3 g daily intake of Bßglucans for 4 weeks.

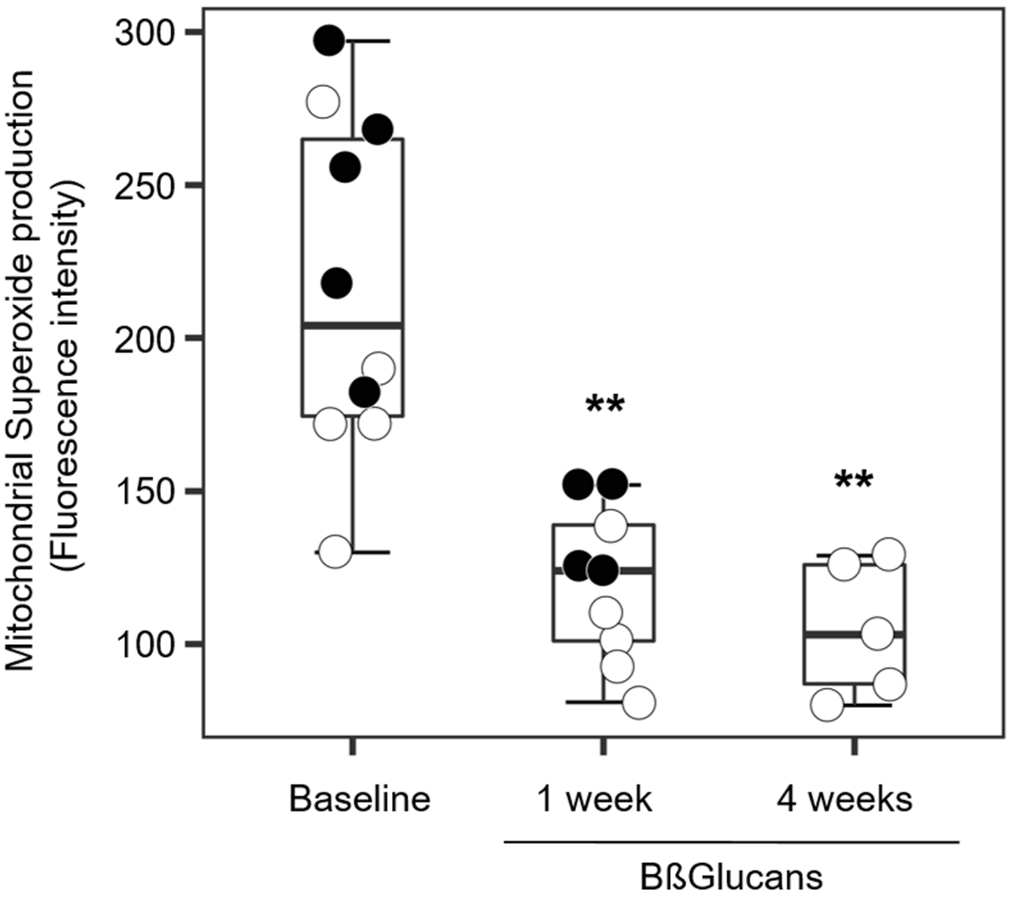

Supplementary Figure 2. Anti-oxidant properties of BßGlucans in immune cells. Mitochondrial superoxide production (expressed as percentage of baseline conditions) by Raw264.7 cells exposed for 16 hours to vehicle (CONTROL), 100μg/mL BßGlucans (BßGlucans), 5μg/mL LPS (LPS) or the combination (LPS+BßGlucans). Bars and error bars represent mean±SD from three independent experiments performed in triplicate per experimental condition. **p<0.01 vs. CONTROL; #p<0.01 vs. LPS.

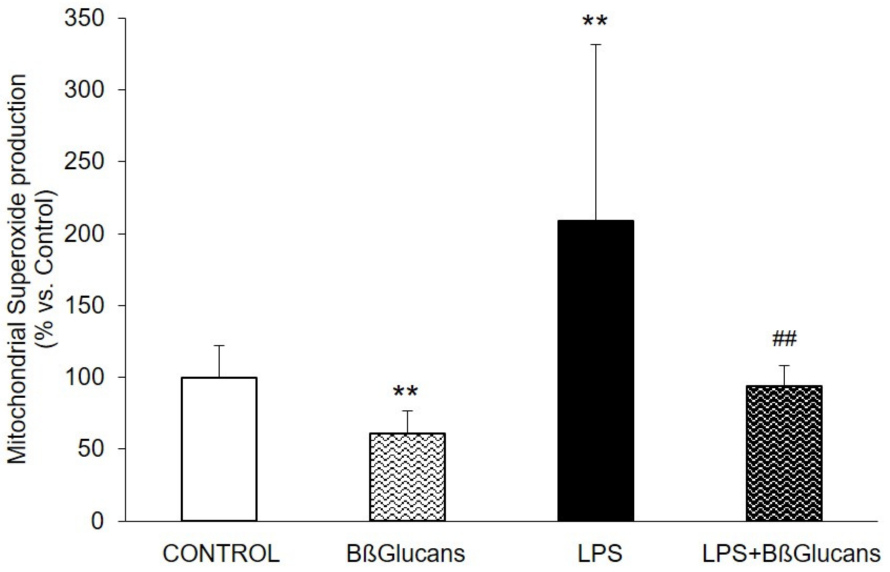

Supplement: Supplementary file 1 — Supplementary information [file 41598_2019_54306_MOESM1_ESM.pdf]
